# On the Present State of Therapeutical Inquiry

**Published:** 1846-04

**Authors:** 


Art. VI.—*On the present State of Therapeutical Enquiry.* By James Arnott, m.d.—*Brighton*, 1845. 12mo. pp. 57; with an Appendix.

Dr. James Arnott first takes occasion to lament the great difficulty the therapeutical discoverer experiences in obtaining a notice. This he attributes principally to the frequent disappointments practitioners experience in the use of new and much praised remedies; the remedies being, in fact, inadequate, because recommended from false analogies or imperfect observation. Dr. Arnott then proceeds to consider, firstly, the principal sources of therapeutical knowledge; secondly, to what degree medical theory has in this respect proved useful; and thirdly, the present defective mode of reporting the results of medical experience, and how it may be amended.

Under the first head, Dr. Arnott indicates the principal sources of therapeutical knowledge to be,—the accidental observation of the effects of remedies; the direct calls of instinct for some remedial means, as in wounds, &c., from mechanical injuries; the artistic or technical perfection of remedies thus discovered; and lastly, the analogy between the observed action of certain medicines by which the one has been substituted for the other. Dr. Arnott gives various interesting examples of remedies from these various sources.

With regard to the influence which medical theories have exercised on the progress of therapeutics, Dr. Arnott expresses an unfavorable opinion. If the explanation of the morbid condition, he observes, rest solely on mechanical or chemical principles, the appropriate remedy may originate from such explanation; but what is called the theory of disease usually implies a consideration of the laws of vitality, and, in this sense of the term, it would be easier to show the mischief which theory has occasioned, than to collect the instances of its direct advantage. It has been the source of many dangerous practices, and by the prejudices it has excited, and the conventionalism and routinism it has developed, the practitioner has been prevented acquiring and adopting better plans of treatment. Dr. Arnott thinks that medical prophylaxis has been more advanced of late years than therapeutics. In this opinion we fully concur, and we think that it may be taken as a strong, and, indeed, irresistible proof, of the great advance made in medical science and art in our own times.

Theory has, however, its value; indeed all our greatest practitioners, whether physicians or surgeons, have been theoretical. The faculty to discover necessarily implies a speculative mind. Now a speculative mind is in fact one which collects and meditates on facts, if so be that it be rightly trained. It is only absurdly speculative when it leaves facts altogether to build theories on hypotheses—a series of unsubstantial erections which vanish into nothingness so soon as the speculator is beckoned from his airy height to the regions of plain common sense and utility. A truly practical man is he who continually endeavours to *adapt* his speculative ideas to the cases of disease that come daily under his notice.

Now we really must give Dr. Arnott the credit of being a speculative man of this class. In his little book the practitioner will find a closely-packed repository of useful inventions. Many of these are eminently ingenious, and based on sound principles. It is, however, in medico-hydraulical mechanics that Dr. James Arnott most distinguishes himself, and we have various ingenious modes for using fluids in the treatment of disease, whether for the dilatation, compression, or regulation of the temperature of parts.

We cannot undertake even to enumerate the different ingenious uses to which fluids, and especially water, are applied by Dr. James Arnott, but assure the practitioner, that the outlay of the trifle which the little book may cost, will be a most excellent expenditure of capital. In addition to the description of various new therapeutical methods, the appendix is more especially devoted to improvements in the obstetric art, many of which are worthy the accoucheur's consideration. Invention seems characteristic of clan Arnott. The present author treads the same path—though at some distance behind his more famous brother Neill.

We would suggest to Dr. James Arnott the expediency of enlarging and somewhat altering the plan of his work in another edition, by noticing at greater length, and on a wider scale, the strictly medical part of his subject. We hope the free-spoken article in our last number may encourage him to tread less gingerly on the toes of ancient prejudices.

